# Antidiabetic Effects of Quercetin and Silk Sericin in Attenuating Dysregulation of Hepatic Gluconeogenesis in Diabetic Rats Through Potential Modulation of PI3K/Akt/FOXO1 Signaling: In Vivo and In Silico Studies

**DOI:** 10.3390/jox15010016

**Published:** 2025-01-19

**Authors:** Heba M. Abdou, Ghada M. Abd Elmageed, Hussein K. Hussein, Imane Yamari, Samir Chtita, Lamia M. El-Samad, Mohamed A. Hassan

**Affiliations:** 1 Department of Zoology, Faculty of Science, Alexandria University, Alexandria 21321, Egypt; heba.abdu@alexu.edu.eg (H.M.A.); ghada.mahmoud_pg@alexu.edu.eg (G.M.A.E.); h.khamis@alexu.edu.eg (H.K.H.); lamya.moustafa@alexu.edu.eg (L.M.E.-S.); 2 Laboratory of Analytical and Molecular Chemistry, Faculty of Sciences Ben M’Sik, Hassan II University of Casablanca, Casablanca P. O. Box 7955, Morocco; imane.yamari-etu@etu.univh2c.ma (I.Y.); samir.chtita@univh2c.ma (S.C.); 3 Protein Research Department, Genetic Engineering and Biotechnology Research Institute (GEBRI), City of Scientific Research and Technological Applications (SRTA-City), Alexandria 21934, Egypt

**Keywords:** type 2 diabetes mellitus, quercetin, silk sericin, hepatic gluconeogenesis, caspase-3, PI3K/Akt/FOXO1 signal

## Abstract

Type 2 diabetes mellitus (T2DM) is an intricate disease correlated with many metabolic deregulations, including disordered glucose metabolism, oxidative stress, inflammation, and cellular apoptosis due to hepatic gluconeogenesis aberrations. However, there is no radical therapy to inhibit hepatic gluconeogenesis disturbances yet. We thus sought to probe the effectiveness and uncover the potential mechanism of quercetin (QCT) and silk sericin (SS) in mitigating hyperglycemia-induced hepatic gluconeogenesis disorder, which remains obscure. Administration of QCT and SS to diabetic male albino rats markedly restored the levels of glucose, insulin, advanced glycation end-products (AGEs), liver function enzymes, alpha-fetoprotein (AFP), globulin, and glycogen, in addition to hepatic carbohydrate metabolizing enzymes and gluconeogenesis in comparison with diabetic rats. Furthermore, treatment with QCT and SS modulated hepatic malondialdehyde (MD), reduced glutathione (GSH), superoxide dismutase (SOD), catalase (CAT), glutathione peroxidase (GPx), nitric oxide, tumor necrosis factor-alpha (TNF-α), and interleukin-1β (IL-1β), in addition to serum interleukin-6 (IL-6) and cyclooxygenase-2 (COX-2), implying their effectiveness in safeguarding cells against oxidative impairment and inflammation. Remarkably, QCT and SS treatments led to the upregulation of expression of phosphatidylinositol 3-kinases (PI3K), phospho-Akt (p-Akt), and forkhead box-O1 (FOXO1) genes in hepatic tissues compared to diabetic rats, orchestrating these singling pathways for curtailing hyperglycemia and pernicious consequences in hepatic tissues. Importantly, immunohistochemical investigations exhibited downregulation of caspase-3 expression in rats treated with QCT and SS compared to diabetic animals. Beyond that, the histopathological results of hepatic tissues demonstrated notable correlations with biochemical findings. Interestingly, the in silico results supported the in vivo findings, showing notable binding affinities of QCT and SS to PI3K, GPx, and TNF-α proteins. These results imply that QCT and SS could mitigate oxidative stress and inflammation and regulate hepatic gluconeogenesis in diabetic rats. However, QCT revealed greater molecular interactions with the studied proteins than SS. Overall, our results emphasize that QCT and SS have significant therapeutic effects on attenuating hyperglycemia-induced hepatic gluconeogenesis, with QCT showing superior effectiveness.

## 1. Introduction

Insulin resistance is the primary causal factor for hyperglycemia and compensatory hyperinsulinemia [1]. The liver, a collection of insulin-sensitive tissues, is one of the vital organs vulnerable to oxidative stress engendered by hyperglycemia. It is believed that oxidative stress impairs liver tissue. As a consequence, protein, carbohydrate, and lipid metabolism could be disrupted, instigating inflammatory cascades [2,3]. Oxidative stress (OS) and inflammatory reactions incite diabetes mellitus (DM) deterioration by damaging the body [4].

Similarly, the liver has a vital function in glucose metabolism. The liver is indispensable for modulating glucose synthesis and deposition, which is essential for gluconeogenesis and glycogen synthesis [5]. Type 2 diabetes mellitus (T2DM) is a complicated disease defined by many metabolic abnormalities, such as impaired glucose metabolism and an aberrant hepatic insulin signaling pathway [6]. Furthermore, lipid irregularities are common in individuals with DM on account of insulin resistance or metabolic disruptions that adversely impact crucial enzymes and relevant pathways involved in lipid metabolism, provoking diabetic dyslipidemia [7]. This symptom is often characterized by elevated serum cholesterol and reduced HDL-cholesterol and triglycerides [8].

It is believed that the insulin receptor-mediated signaling pathway is vital for blood glucose modulation. In this context, the phosphatidylinositol 3-kinase/protein kinase B (PI3K/Akt) signaling pathway is widely recognized as the leading signal transmission mechanism and exerts significant regulatory control over gluconeogenesis [9]. It is also recognized that the PI3K/Akt pathway plays a key role in protecting cells against oxidative impairment and inflammation [10,11]. Additionally, it is reported that G6-Pase and PEPCK are the crucial enzymes that control gluconeogenesis [12], while forkhead box-O1 (FOXO1) regulates insulin sensitivity. Critically, insulin resistance is frequently associated with a reduction in PI3K/Akt signals, leading to an increase in hepatic glucose metabolism. This is caused by an upregulation of PEPCK and G6Pase expression, which is governed by FOXO1 [5].

In light of this, numerous medicinal plants were applied for DM treatment because of their safety, low toxicity, and enhanced accessibility rather than synthetic drugs. Quercetin (QCT) is a flavonoid, which is broadly occurring in propolis and medicinal plants, in addition to fruits and vegetables, particularly onions, broccoli, grapes, apples, green tea, and red wine [13,14,15]. It is also found in various seeds, flowers, and leaves, as well as in algal extracts [16,17,18]. In biomedical applications, it was revealed the inhibitory effects of QCT on liver damage and inflammation, as well as the effectiveness of treating liver fibrosis in animal models related to several species [19]. Moreover, it has been demonstrated the efficacy of QCT in counteracting the pernicious influences of pancreatic islets in rats due to DM [20,21]. This occurs by modulating glucose homeostasis, lessening insulin resistance, abating oxidative damage, and precluding cell death. QCT safeguards the liver against a variety of liver illnesses and has beneficial effects on the metabolism of glucose and lipids. Therefore, QCT has the potential to treat glucose and lipid metabolism deregulations in individuals with DM [22].

Additionally, insect-derived drugs have gained profuse attention due to their therapeutic efficacy in managing various diseases [23]. With their diverse composition and exceptional pharmaceutical properties against a wide range of diseases and infections, insects possess immense potential for the development of innovative and efficient drugs [23,24,25]. Among therapeutics purified from insects, silk sericin (SS) is a biopolymer protein extracted from the silkworm *Bombyx mori*, which is non-toxic with low immunogenicity and naturally present in nature [26,27,28,29]. Several therapeutic properties of sericin have been demonstrated, including antioxidant, anti-inflammatory, anticancer, tyrosinase inhibitory, and antimicrobial properties, as well as safeguarding the liver and stomach from alcohol-induced impairment [27,30,31,32]. In addition, a previous report showed that dietary SS reduced triglycerides and cholesterol concentrations in rats fed a high-fat diet [33].

Even though QCT and SS are recognized as significant therapies in counteracting detrimental effects incited by DM, their efficacy and underlying mechanisms in treating the malfunction of hepatic gluconeogenesis due to T2DM remain obscure. In this context, our previous studies have demonstrated the neurotherapeutic effects of QCT extracted from *Trifolium alexandrinum* (TA) on regulating diabetes-induced cerebral cortical impairment and enhancing wound repair and skin tissue restoration in diabetic rats [34,35]. Therefore, this investigation aims to assess the impact of QCT extracted from *T. alexandrinum* (TA) and SS derived from silkworm *B. mori* on regulating T2DM in male rats. Our study provides substantial evidence supporting their efficacy as anti-diabetic, antioxidant, and anti-inflammatory treatments. Additionally, we evaluated the effectiveness of QCT and SS in coordinating the PI3K/Akt/FOXO1 signaling pathway. Furthermore, we examined the histopathological and immunohistochemical alterations of liver tissues to demonstrate the ability of QCT and SS to restore hepatic structures. Moreover, in silico studies of QCT and SS were carried out, including molecular docking simulations and protein-protein interactions, to examine their interactions with PI3K, a crucial regulator protein in glucose metabolism, as well as glutathione peroxidase (GPx) and tumor necrosis factors to verify their antioxidant and anti-inflammatory characteristics.

## 2. Materials and Methods

### 2.1. Chemicals

Streptozotocin (STZ, Cat. No. 18883-66-4) was provided by Sigma-Aldrich Chemical Company (Rockville, MD, USA), while silk sericin powder (Cat. No. S5201-5G) from *Bombyx mori* (silkworm) was supplied by Sigma-Aldrich, Saint-Quentin-Fallavier, France. All additional reagents utilized in this study met analytical standards.

### 2.2. Quercetin Extraction

Quercetin utilized in the current research was isolated from clover (*Trifolium alexandrinum* Linn) following multistep extraction procedures as described in our previous report. The extract was purified with ethanol and silica gel chromatography, followed by capillary electrophoresis to isolate QCT, and then identified employing high-performance liquid chromatography (HPLC) [35].

### 2.3. Animal Care

All animal studies and relevant procedures were accomplished in compliance with the ARRIVE guidelines (https://arriveguidelines.org, accessed on 17 September 2021) and ethically approved by the Institutional Animal Care and Use Committee (ALEXU-IACUC), Alexandria University, Egypt (approval number: AU0421923202). The study included adult male *Wistar albino* rats weighing between 150 and 170 g. The animals were housed in conventional cages in a well-ventilated facility, regulated with a 12-h light-dark cycle and a temperature of 22 ± 2 °C. A standard diet was provided ad libitum, along with unrestricted tap water access.

### 2.4. Experimental Design and Diabetic Induction

**Figure 1:**
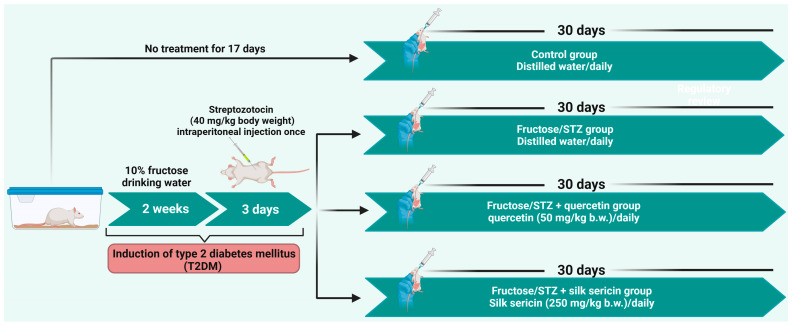
A schematic illustration depicts the design and timeline of the in vivo study.

Following acclimation for one week, a total of 24 rats were randomly assigned into two major groups: Group 1 (control group, 6 rats) was provided with distilled water via oral gavage for 4 weeks. Group 2: 18 rats were exposed to diabetes induction. Group 2 rats were given a 10% fructose solution in their drinking water for 2 weeks, followed by intraperitoneally injection with a single dose of STZ, 40 mg/kg body weight (b.w.) to induce T2DM [36,37]. On the third day after STZ injection, the glucose level in the blood was assessed by means of a glucometer. Accordingly, rats with fasting blood glucose higher than 220 mg/dL were defined as diabetic rats [38,39]. Afterward, diabetic rats were assigned into three sub-groups (*n* = 6), including Group 2: untreated diabetic rats Fructose/STZ-treated group, Group 3: Fructose/STZ + quercetin (50 mg/kg b.w./day, orally) [40,41], and Group 4: Fructose/STZ + sericin (250 mg/kg b.w./day, orally) [42,43]. Rats were given quercetin and silk sericin once daily for 30 days. Figure 1 illustrates the experimental design and timeline, including the doses of each drug.

### 2.5. Blood Samples Collection and Tissue Preparation

By the end of the experimental duration, the animals were fasted before being sacrificed under ketamine/xylazine anesthesia. Blood was obtained from the abdominal aorta of fasted rats. After coagulation, specimens were clarified at 3000 rpm for 10 min, followed by preservation of the supernatants at −80 °C for subsequent investigations. The hepatic tissues were dissected and washed in a chilled saline solution. Small portions of hepatic tissues were obtained and maintained at −80 °C for RNA extraction. Other parts of liver tissues were homogenized and clarified at 8000 rpm for 15 min, followed by maintaining the supernatants at −80 °C for biochemical assays. On the other hand, other slices of hepatic tissues were immediately fixed in 10% formalin for histopathological and immunohistochemical inspections.

### 2.6. Biochemical Examinations

#### 2.6.1. Estimation of Serum Glucose, Insulin, and AGEs

Serum glucose was measured using a commercially available diagnostic kit (Bio Diagnostic Co., Giza, Egypt). Insulin determination was conducted with the help of the enzyme-linked immunosorbent assay (ELISA) kit (Cat No. ERINS, Invitrogen, Waltham, MA, USA). The concentration of advanced glycation end products (AGEs) was determined by means AGE competitive ELISA kit (Cat. No. STA-817-T, Cell Biolabs’ OxiSelect™, San Diego, CA, USA). All determinations were conducted in six replicates.

#### 2.6.2. Evaluation of Lipid Profile

Total cholesterol (TC) and triglyceride (TG) were appraised utilizing respective kits provided by Bio Diagnostic Co. (Cat No. CH1220 and TR2030, Bio Diagnostic Co., Giza, Egypt), respectively. HDL-cholesterol and LDL-cholesterol were estimated utilizing standard kits (Cat No. CH1230 and CH1231, Bio Diagnostic Co., Giza, Egypt), respectively. VLDL-cholesterol was detected according to the protocol of Wilson et al. [44]. All examinations were preformed six times.

#### 2.6.3. Evaluation of Liver Function Enzymes, Albumin, Globulin, Total Protein, Bilirubin, AFP in Serum, and Hepatic Glycogen

Aspartate aminotransferase (AST; Cat No. AS1061), alanine transaminase (ALT; Cat No.1031), alkaline phosphatase (ALP; Cat No. AP1020), total protein (Cat No. TP2020), albumin (Cat No. AB1010) and total bilirubin (BR; Cat No.1111) were detected in serum by respective kits provided by Bio Diagnostic Co., Giza, Egypt, following the protocols supplied by the manufactures. Globulin was assessed by following equation:

Globulin (g/dL) = total proteins (g/dL) − albumin (g/dL)

Alpha-fetoprotein was evaluated by a commercial kit (AFP; Cat. No. RK03475, AB clonal, Woburn, MA, USA). Hepatic glycogen levels were assessed by a rat glycogen kit (Cat No. ab282931, Abcam Co., Cambridge, UK). A total of six replications were carried out for each assay.

#### 2.6.4. Assessment of Hepatic Carbohydrate Metabolizing Enzymes

Using ELISA kits, hepatic hexokinase (HK; Cat No. E4600-100, Biovision, Milpitas, CA, USA), hepatic glucokinase activity (GK; Cat No. E4598-100, Biovision, Milpitas, CA, USA), hepatic glucose-6-phosphate dehydrogenase (G6Pd; Cat No. K540-100, Biovision, Milpitas, CA, USA), and hepatic glucose-6-phosphate (G6P; Cat No. E-BC-K011-M, Elab Science, Houston, TX, USA) were assessed. All determinations were accomplished in six replicates.

#### 2.6.5. Estimation of Oxidative Stress Markers and Antioxidant Activities

To estimate malondialdehyde and reduced glutathione levels in hepatic homogenates of rats, MDA (Cat No. MD2529) and GSH (Cat No. GR2511) kits from Bio Diagnostic Co., Giza, Egypt were used. Furthermore, we assessed superoxide dismutase, glutathione peroxidase, and catalase activities in homogenates of liver tissues using kits from Bio Diagnostic Co., Giza, Egypt (SOD; SD2521), (GPx; GP2524), and (CAT; CA2517), respectively. The nitric oxide level was assessed calorimetrically employing a respective kit (Cat No. NO25-33, Bio Diagnostic Co., Giza, Egypt). All determinations were conducted in six replicates.

#### 2.6.6. Determination of Inflammatory Biomarkers

Using ELISA kits, the liver concentrations of tumor necrosis factor-alpha (TNF-α; Cat No. MBS2507393, MyBioSource Co., San Diego, CA, USA), interleukin-1β (IL-1β; Cat No. BMS630, Thermo Fisher Scientific, Agawam, MA, USA), interleukin 6 (IL-6; Cat No. MBS726707, MyBioSource Co., San Diego, CA, USA), and cyclooxygenase 2 (COX-2; Cat No. ab300668, Abcam Co., Cambridge, UK) were quantified in hepatic homogenates. Each examination was performed in six replicates.

#### 2.6.7. Quantitative Real-Time PCR (qRT-PCR) for PI3K, Akt, and FOXO1 Genes

Total RNA was isolated from hepatic tissues via the RNA-Spin Kit (Biovision Co., San Jose, CA, USA) following procedures furnished by the manufacturer. The expressions of phospho-Akt (p-Akt), phosphatidylinositol 3-kinases (PI3K) and forkhead box-O1 (FOXO1) genes were evaluated adopting quantitative reverse transcription-PCR (qRT-PCR) by means of a real-time SYBR Green gene expression test kit (Cat. No. 204243, QIAGEN, Hilden, Germany). The extracted RNA was utilized to produce cDNA using a cDNA synthesis kit. The mRNA expression of the genes, along with the reference gene, were evaluated via gene-specific SYBR Green-based QuantiTect^®^ Primer assays from QIAGEN, Hilden, Germany. The qPCR assay was conducted using specific primers as follows: PI3K (Accession number: NM_001371300.2), F: 5′-CCTGGTAACTGCAACACTTC-3′ and R: 5′-AACGAATTCAAACCTACCCG-3′; Akt (Accession number: NM_ 033230.3), F: 5′-TAGCCATTGTGAAGGAGGGC-3′ and R: 5′-TCAGCTGACATTGTGCCACT-3′; FOXO1 (Accession number: XM_039103268.1), F: 5′-TCATCCAATTGGTCTTGTGG-3′ and R: 5′-GTGTTTGCCTGTCTACCTTT-3′; and β-Actin (Accession number: XM_039089807.1), F: 5′-ATGTGGCTGAGGACTTTGATT-3′ and R: 5′-ATCTATGCCGTGGATACTTGG-3′.

The optimal annealing temperature and duration were determined for each combination of primer and template, while β-actin served as an endogenous control for the normalization of expression levels. The experiment was replicated five times, and the 2^−ΔΔCt^ approach was employed to compute mRNA expression [45].

### 2.7. Histopathological Examination

To probe the histological attributes of hepatic tissues from different animal groups, the tissues were immersed in 10% neutral-buffered formalin and then dehydrated in different ethanol concentrations. After embedding in paraffin wax, hepatic tissues were prepared by slicing into 5 µm sections before being stained with hematoxylin and eosin (H&E) and probed by a light microscope (Olympus CX31, Tokyo, Japan).

### 2.8. Immunohistochemical Investigation

Hepatic tissues embedded in paraffin were deparaffinized with xylene and then gradually rehydrated by ethanol solutions. Afterward, the samples were retrieved by heating in 1× target retrieval solution (pH 6.0, DAKO, Glostrup, Denmark), followed by blocking with a peroxidase-blocking solution for 15 min. After washing with PBS buffer, the hepatic sections were blocked with serum block solution for 10 min prior to being incubated overnight at 4 °C with the primary antibody caspase-3 (1:100, D175, Cell Signaling Technology, Beverly, MA, USA). Following washing with PBS buffer, the hepatic sections were treated with AEC (3-Amino-9-ethylcarbazole) substrate chromogens, followed by incubation with a horseradish peroxidase (HRP)-labeled micropolymer conjugated with secondary antibody (MP-7451, anti-rabbit IgG; Vector Labs, Burlingame, CA, USA) for 1 h [46]. Subsequently, the slides were washed, stained with hematoxylin as a secondary stain, and then probed employing a light microscope (Olympus CX31, Tokyo, Japan).

### 2.9. In Silico Screening and Docking Studies

To elucidate the molecular mechanisms underlying the antidiabetic influences of QCT and SS, we conducted in silico studies, including molecular docking simulations for QCT and protein-protein interaction for SS. These computational approaches were employed to explore the interactions of QCT and SS with a key protein, PI3K, involved in glucose metabolism, in addition to GPx and TNF-α implicated in antioxidant and anti-inflammatory activities of both compounds.

#### 2.9.1. Molecular Docking of Quercetin

Molecular docking simulations were performed using AutoDock 1.5.7 [47], to assess quercetin’s binding affinity with PI3K, GPX, and TNF-α. The preparation involved optimizing the protein structures (PI3K, GPX, and TNF-α) obtained from the PDB database (IDs: 1E90 (https://www.rcsb.org/structure/1E90, accessed on 17 December 2024), 2F8A (https://www.rcsb.org/structure/2F8A, accessed on 17 December 2024), 6OP0 (https://www.rcsb.org/structure/6OP0, accessed on 17 December 2024), respectively, using Swiss-PDB Viewer 4.1.0 [48] and we refined any missing atoms or residues. The ligand (QCT) was then optimized using Avogadro 1.2.0 [49], and the grid box for docking was identified.

#### 2.9.2. Protein-Protein Docking of Silk Sericin

Protein-protein docking studies were also conducted against the similar target proteins (PI3K, GPx, and TNF-α) employing the HADDOCK 2.4 web server [50,51,52], leveraging its clustering algorithm to examine the interactions between SS and investigate potential binding mechanisms. The active sites were predicted using the MetaPPISP tool (https://pipe.rcc.fsu.edu/meta-ppisp.html, accessed on 17 December 2024) (Table 1) [53].

### 2.10. Statistical Analysis

All biochemical determinations were implemented in six replicates except qRT-PCR, which was replicated five times. Before commencing the statistical analysis, we checked the validity of the data following a Kolmogorov-Smirnov test. Afterwards, the statistical analysis was conducted adopting one-way analysis of variance (ANOVA) associated with Tukey’s post-hoc test for multiple comparisons between different experimental groups employing SPSS (Version 25, IBM Software, Armonk, NY, USA) and GraphPad Prism (Version 8, GraphPad Software Inc., Boston, MA, USA). All result values are presented as mean ± SD, and significant differences were determined at *p* ≤ 0.05, whereas the high significant differences were considered at *p* ≤ 0.01, *p* ≤ 0.001, and *p* ≤ 0.0001.

## 3. Results

### 3.1. Evaluation of Glucose, Insulin and AGEs in Serum

**Figure 2:**
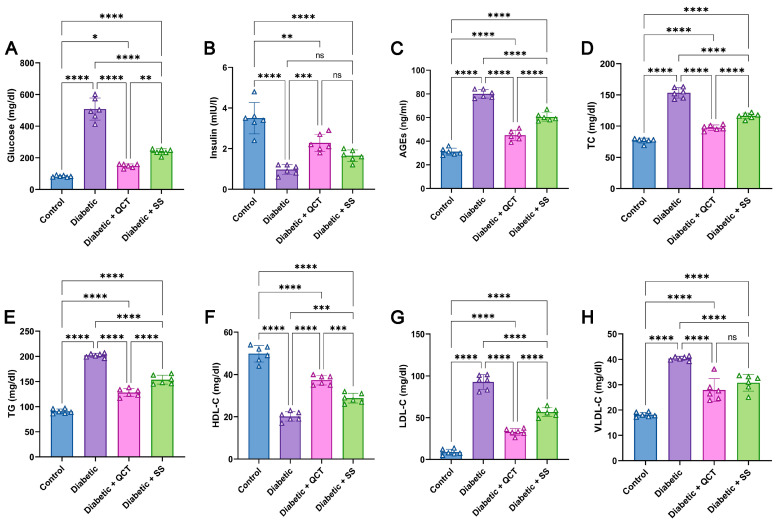
Assessment of (**A**) glucose, (**B**) insulin, and (**C**) advanced glycation end-products (AGEs) in serum of different animal groups. Estimation of lipid profile in animal serum, including (**D**) total cholesterol (TC), (**E**) triglycerides (TG), (**F**) HDL-cholesterol (HDL-C), (**G**) LDL-cholesterol (LDL-C), and (**H**) VLDL-cholesterol (VLDL-C). All measurements are depicted as mean ± SD (n = 6) (**** *p* < 0.0001, *** *p* < 0.001, ** *p* < 0.01, and * *p* < 0.05, while ns denotes non-significant differences).

The concentrations of glucose and AGEs in serum were considerably heightened in Fructose/STZ diabetic rats in relation to control animals as depicted in Figure 2A,C. On the other hand, Figure 2B illustrates a noticeable decrease in serum insulin concentration in diabetic animals compared to untreated rats. By contrast, the animal groups administered QCT and SS exhibited noticeable restoration of glucose, AGEs, and insulin concentrations to normal levels compared with the Fructose/STZ diabetic group.

### 3.2. Assessment of Serum Lipid Profile

It can be observed from Figure 2D–H that the Fructose/STZ diabetic group showed marked augmentations in the concentrations of TC, TG, LDL-C, and VLDL-C in serum, while the HDL-C level was substantially diminished. On the contrary, diabetic rats provided with QCT and SS manifested noticeable ameliorations in all lipid profile parameters compared to the Fructose/STZ diabetic group.

### 3.3. Assessment of Liver Function Enzymes, Albumin, Globulin, Total Protein, Bilirubin, AFP in Serum, and Hepatic Glycogen

**Figure 3:**
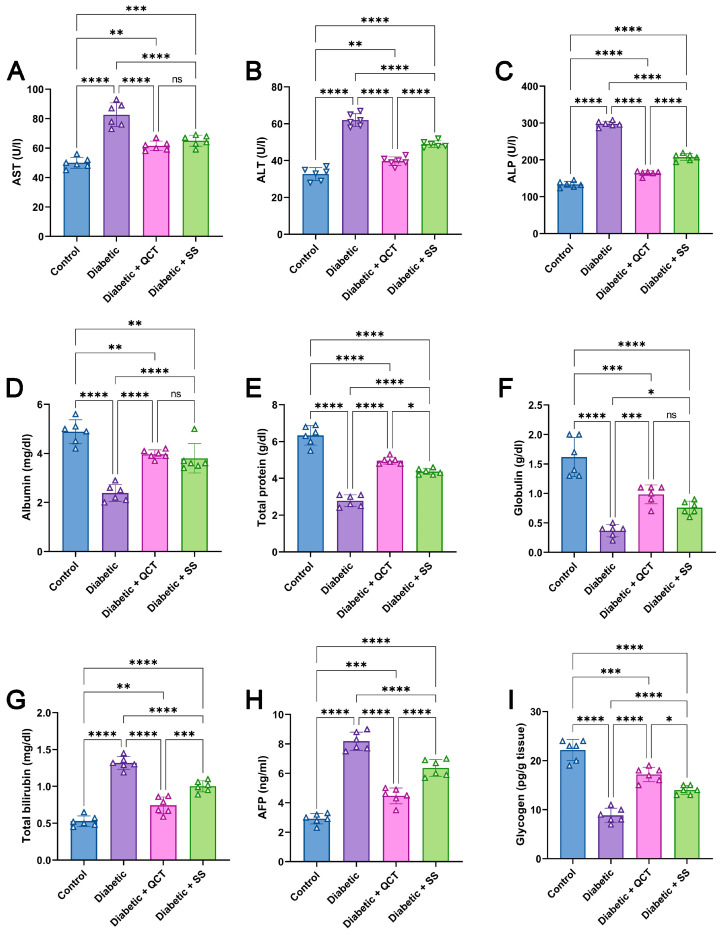
Estimation of (**A**) aspartate aminotransferase (AST), (**B**) alanine transaminase (ALT), and (**C**) alkaline phosphatase (ALP), (**D**) albumin, (**E**) total protein, (**F**) globulin, (**G**) total bilirubin, and (**H**) Alpha-fetoprotein (AFP) in serum of different animal groups. Evaluation of (**I**) hepatic glycogen in animal groups. All measurements are depicted as mean ± SD (n = 6). (**** *p* < 0.0001, *** *p* < 0.001, ** *p* < 0.01, and * *p* < 0.05, while ns denotes non-significant differences).

It can be seen in Figure 3A–C that Fructose/STZ diabetic rats exhibited noticeable elevations in AST, ALT, and ALP activities compared with control animals. However, the concentrations of albumin, globulin, and total protein were significantly lower as displayed in Figure 3D–F. Furthermore, Figure 3G–I exhibit that the diabetic rats demonstrated heightened levels of total bilirubin and AFP, along with a marked diminution in liver glycogen level in comparison with control animals. As a result of rats being administered QCT and SS, AST, ALT, and ALP activities dwindled. Besides, albumin, total protein, and globulin were markedly increased in diabetic animals supplied with QCT and SS compared with Fructose/STZ diabetic rats. While matched to normal rats, QCT and SS in the Fructose/STZ group also revealed a substantial reduction in total bilirubin and AFP, associated with a notable rise in liver glycogen level.

### 3.4. Evaluation of Hepatic Carbohydrate Metabolizing Enzymes

**Figure 4:**
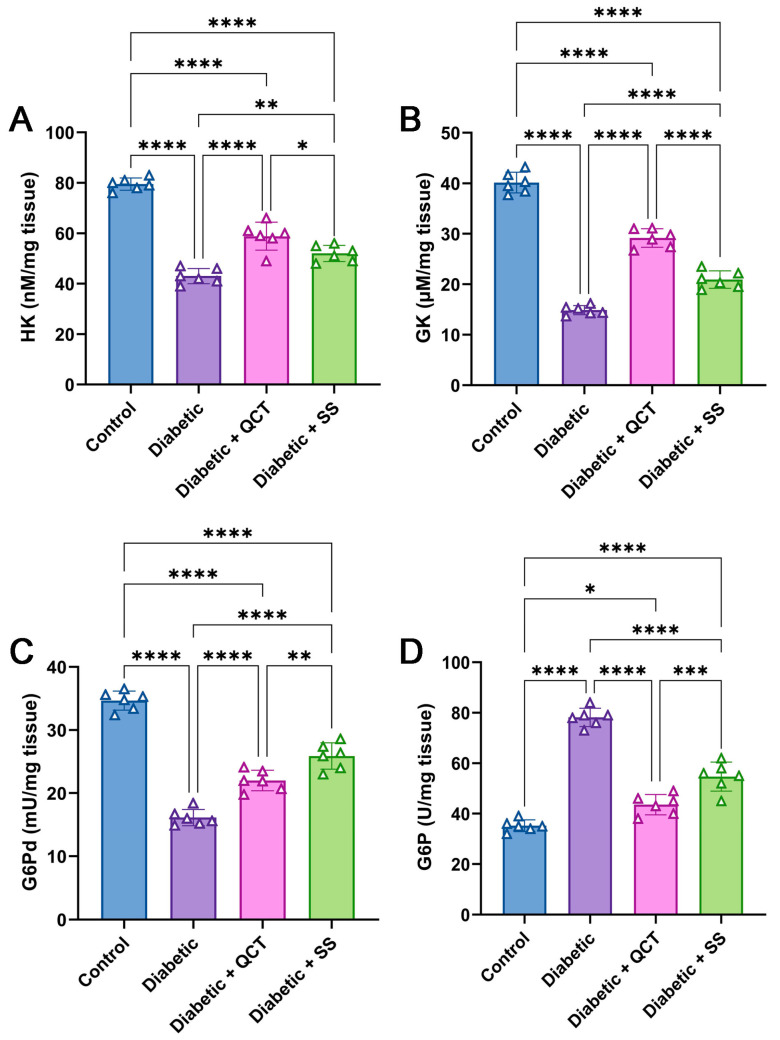
Evaluation of (**A**) hexokinase (HK), (**B**) glucokinase (GK), (**C**) glucose-6-phosphate dehydrogenase (G6Pd), and (**D**) glucose-6-phosphate (G6P) in hepatic tissues from different animal groups. All measurements are shown as mean ± SD (n = 6). (**** *p* < 0.0001, *** *p* < 0.001, ** *p* < 0.01, and * *p* < 0.05).

It is evident from the data in Figure 4A–C that hepatic HK, GK, and G6Pd activities were substantially lessened in the Fructose/STZ-induced group compared with control animals. In addition, the rats in the Fructose/STZ-induced diabetes group showed a noticeable rise in G6P activity in hepatic tissues in relation to the control rats as shown in Figure 4D. In contrast, diabetic rats receiving QCT and SS demonstrated a notable increase in hepatic HK, GK, and G6Pd activity in contrast to Fructose/STZ-induced rats. Furthermore, a substantial drop in hepatic G6P activity was detected in QCT and SS-treated animals related to diabetic rats.

### 3.5. Assessment of Oxidative Stress Parameters in Liver Tissue

**Figure 5:**
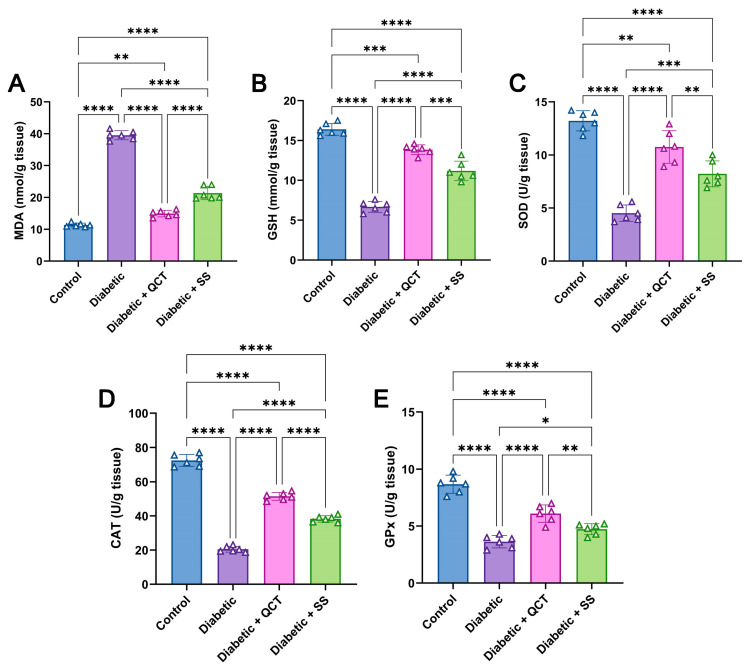
Evaluation of (**A**) malondialdehyde (MD) level, (**B**) reduced glutathione (GSH) level, (**C**) superoxide dismutase (SOD) activity, (**D**) catalase (CAT) activity, and (**E**) glutathione peroxidase (GPx) activity in hepatic tissues from different animal groups. All result values are exhibited as mean ± SD (n = 6). (**** *p* < 0.0001, *** *p* < 0.001, ** *p* < 0.01, and * *p* < 0.05).

Considering the analysis of oxidative stress factors, Figure 5A depicts that induction of diabetes markedly raised MDA level in liver tissues compared with control animals. Furthermore, the Fructose/STZ diabetic group showed a lower level of GSH, in addition to reduced SOD, CAT, and GPx activities in comparison with the untreated animals as displayed in Figure 5B–E. Conversely, the QCT and SS administration resulted in markedly diminished MDA, in addition to notably increased GSH concentration and SOD, CAT, and GPx activities in relation to Fructose/STZ diabetic rats.

### 3.6. Evaluation of Inflammatory Mediators

**Figure 6:**
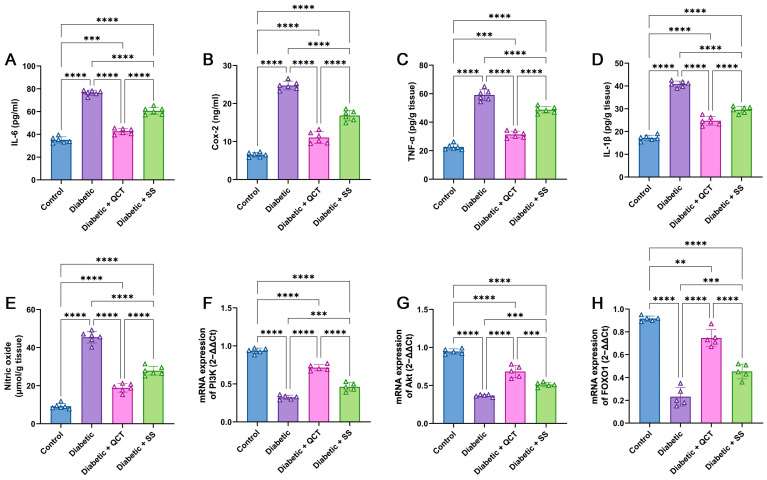
Assessment of (**A**) interleukin-6 (IL-6) and (**B**) cyclooxygenase-2 (COX-2) in serum from rat groups. (**C**) tumor necrosis factor-alpha (TNF-α), (**D**) interleukin-1β (IL-1β), and (**E**) nitric oxide levels in liver tissues from different animal groups. Expression of mRNA of (**F**) phosphatidylinositol 3-kinases (PI3K), (**G**) phospho-Akt (p-Akt), and (**H**) forkhead box-O1 (FOXO1) in liver tissues of different animal groups. All results are shown as mean ± SD (n = 6 for inflammatory biomarkers and n = 5 for qRT-PCR results). (**** *p* < 0.0001, and *** *p* < 0.001, and ** *p* < 0.01).

Figure 6A–E show that concentrations of IL-6 and COX-2 in serum, in addition to TNF-α, IL-1β, and NO in liver homogenates from Fructose/STZ diabetic rats were considerably augmented compared with control rats. By contrast, QCT and SS administration resulted in remarkable reductions in all inflammatory parameters compared to diabetic rats, mitigating the inflammation signs.

### 3.7. Assessment of Akt, PI3K and FOXO1 Gene Expressions in Hepatic Tissues

It can be perceived in Figure 6F–H that Akt, PI3K, and FOXO1 genes expression in the liver tissues of Fructose/STZ diabetic rats were considerably downregulated compared with control rats. Alternatively, QCT and SS treatment of Fructose/STZ diabetic animals caused noticeable upregulations in Akt, PI3K, and FOXO1 compared to the Fructose/STZ diabetic group.

### 3.8. Principle Component Analysis (PCA)

**Figure 7:**
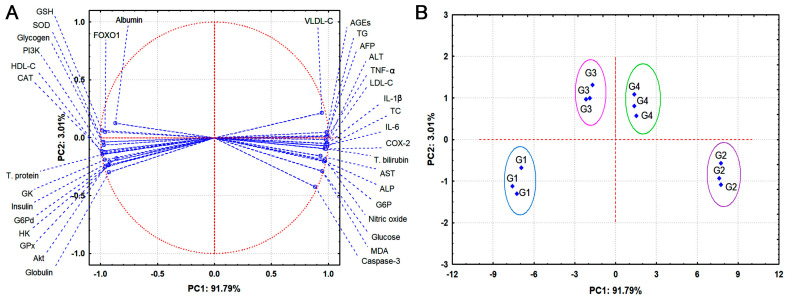
(**A**) Principal component analysis (PCA) of various factors evaluated in this study. (**B**) 2D PCA plot reveals the relationship among different experimental groups. G1: control group, G2: diabetic group, G3: diabetic + quercetin group, and G4: diabetic + silk sericin group.

Figure 7 shows PCA of various factors evaluated in this study to assess their impacts on different animal groups. It is apparent from Figure 6A that PCA explains 94.8% of our findings variation. It is observed that PC1 can explain 91.79% of the differences. This analysis shows the significant influence of PI3K, which could be initially activated by quercetin and silk sericin for further stimulating Akt and FOXO1. Additionally, positive and negative correlations could be perceived between PCA and the different parameters. Figure 6B depicts the relationship among different experimental groups based on the PCA. It shows the emergence of group 1 (control group) on the left side, while group 2 (diabetic group) emerged on the opposite side. This points to the remarkable difference between the control and diabetic groups. Interestingly, the cluster of group 3 (diabetic + quercetin group) shifted to the right side, very close to the control group, implying the significant restoration of various parameters as a result of quercetin treatment. On the other hand, the cluster of group 4 (diabetic + silk sericin group) shifted to the side of the control group, but the cluster exhibited on the left side. Overall, PCA analysis demonstrated that quercetin treatment is more effective than silk sericin at alleviating disorders of different parameters in diabetic rats.

### 3.9. Histopathological Investigation of Hepatic Tissues

**Figure 8:**
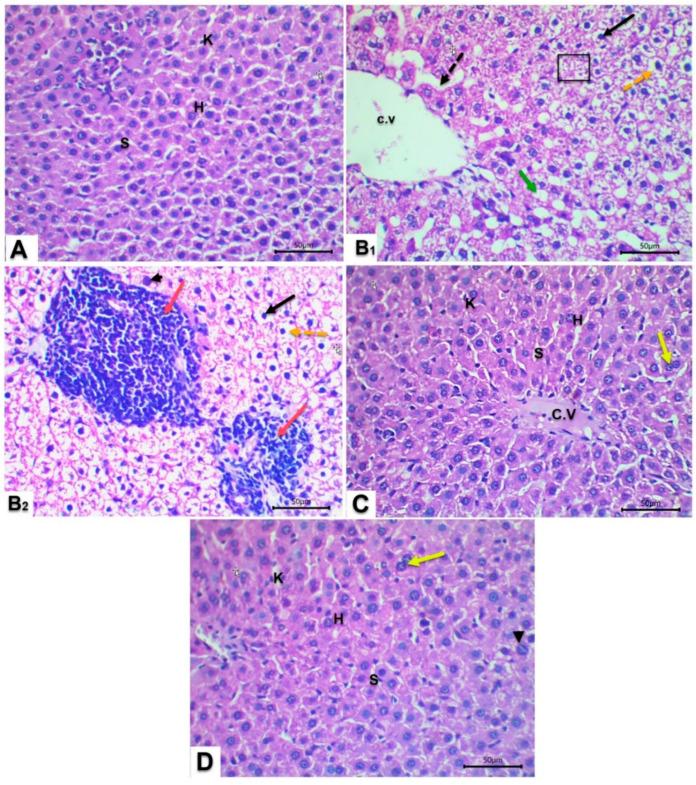
Histopathological observations of hepatic sections from different groups of male rats. (**A**) Control, (**B1**,**B2**) Fructose/STZ diabetic group, (**C**) diabetic rats + QCT group, and (**D**) diabetic rats + SS group. CV, central vein; H, Hepatocytes; K, Kupffer cell; S, blood sinusoid; ballooned hepatocytes (orange dotted arrow), (black dotted arrow) dilation of blood sinusoid, fatty degeneration (black square), cytoplasmic vacuole (green arrow), pyknotic nuclei (black arrow), cellular infiltration (red arrow), binucleated hepatocytes (yellow arrow) and giant nuclei (arrowhead). Haematoxylin and Eosin stain (H&E, ×400 magnification).

Histological examination of the control livers revealed well-defined hepatic lobules with radially arranged hepatocytes surrounding the central vein as delineated in Figure 8A. These hepatocytes showed centrally located, spherical nuclei, and homogenous cytoplasmic staining. Notably, obvious hepatic sinusoids could be perceived. By contrast, liver tissues from Fructose/STZ diabetic animals exhibited critical hepatocellular impairment, characterized by necrosis with pyknotic nuclei, marked dilatation of sinusoidal vasculature, and an increase in Kupffer cells (Figure 8B1,B2). Furthermore, extensive infiltration of inflammatory cells, fatty changes within hepatocytes, expanding degeneration, and a rise in binucleated hepatocytes could be observed.

Administration of either QCT or SS to diabetic rats exhibited a protective effect, mitigating the severity of these lesions and promoting a restoration of normal hepatic architecture as presented in Figure 8C,D. However, both treatment groups showed activation of Kupffer cells and the presence of giant nuclei. Table 2 presents the quantification of lesions observed in histological liver tissues stained with H&E.

### 3.10. Examination of Caspase-3 Immunohistochemistry in Liver Tissues

**Figure 9:**
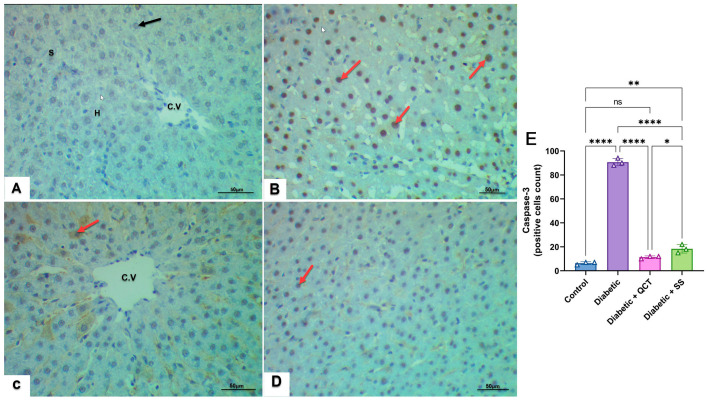
Photomicrographs of sections of the liver tissues from male rats, showing negative immunoreactivity (black arrow) for caspase-3 in control (**A**). Positive expression of caspase-3 (red arrow) in Fructose/STZ diabetic group (**B**). (**C**,**D**) Sections from animals co-treated with QCT + Fructose/STZ and SS + Fructose/STZ, respectively, showed weak immunoreactivity to caspase-3 (×400 magnification). (**E**) Quantification of caspase-3 positively expressed cells in hepatic sections using three slides from each animal group. Results are presented as mean ± SD (n = 3), and **** *p* < 0.0001, ** *p* < 0.01, * *p* < 0.05, whereas ns denotes non-significant difference.

Figure 9A demonstrates that hepatic sections from the control group showed negative caspase-3 immune-reactive cells, while sections from Fructose/STZ diabetic rats revealed positive caspase-3 immune-reactive cells as shown in Figure 9B. In contrast, it is obvious from IHC images that diabetic rats administered QCT or SS demonstrated weak caspase-3 expression as depicted in Figure 9C–E.

### 3.11. Molecular Docking Interactions of Quercetin with PI3K, GPx, and TNF-α

The molecular docking findings revealed significant interactions between QCT and these proteins, with binding energies of −8.1 kcal/mol for PI3K, −6.4 kcal/mol for GPx, and −10.3 kcal/mol for TNF-α as presented in Table 3.

**Figure 10:**
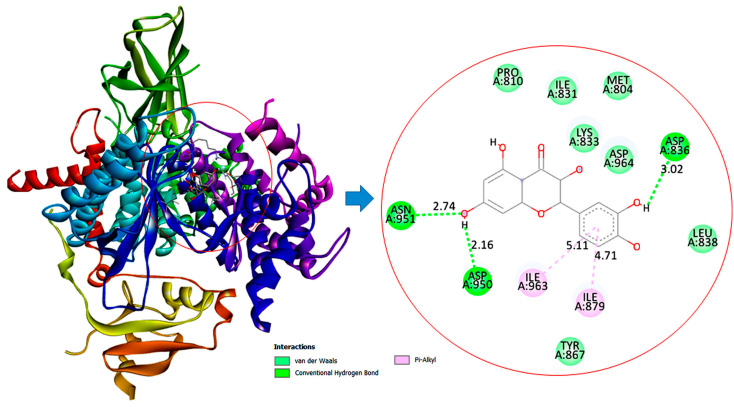
Two and three-dimensional illustrations depict the molecular interaction of quercetin (QCT) with the PI3K active site.

For PI3K, QCT interacts with critical residues like ASN951 (2.74 Å), ASP836 (3.02 Å), and ASP950 (2.16 Å), along with hydrophobic Pi-Alkyl interactions with ILE879 (4.71 Å) and ILE963 (5.11 Å), suggesting it could modulate hepatic gluconeogenesis as portrayed in Figure 10.

**Figure 11:**
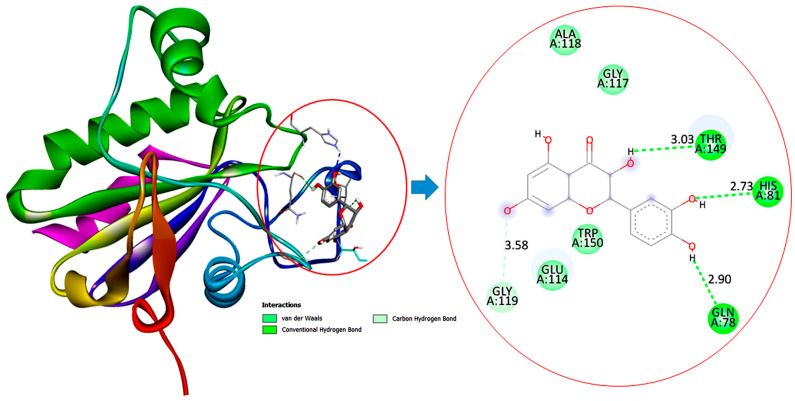
Two and three-dimensional illustrations depict the molecular interaction of quercetin (QCT) with the GPx active site.

Figure 11 demonstrates that GPx interacts with QCT, forming multiple hydrogen bonds with residues such as HIS81 (2.73 Å), GLN78 (2.90 Å), and THR149 (3.03 Å), enhancing its antioxidant activity.

**Figure 12:**
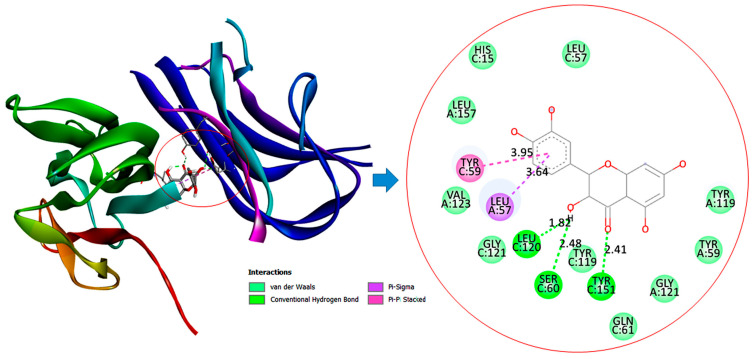
Two and three-dimensional illustrations depict the molecular interaction of quercetin (QCT) with the TNF-α active site.

Concerning interaction with TNF-α, QCT establishes robust hydrogen bonds with SER60 (2.48 Å) and LEU120 (1.82 Å), while hydrophobic interactions with LEU57 (3.64 Å) and TYR59 (3.95 Å) stabilize its binding, indicating its potential to inhibit inflammation as illustrated in Figure 12. These results highlight quercetin’s multifaceted action in mitigating oxidative stress and inflammation, and modulating hepatic gluconeogenesis in diabetic conditions.

### 3.12. Protein-Protein Interactions of Silk Sericin with PI3K, GPx, and TNF-α

The docking results showed that SS interacts with these proteins at specific active sites. The HADDOCK scores for these interactions were −69.3 for PI3K, −75.3 for GPx, and −60.4 for TNF-α with RMSD values of 0.5 ± 0.3, 0.6 ± 0.3, and 0.6 ± 0.6, respectively, as represented in Table 4.

**Figure 13:**
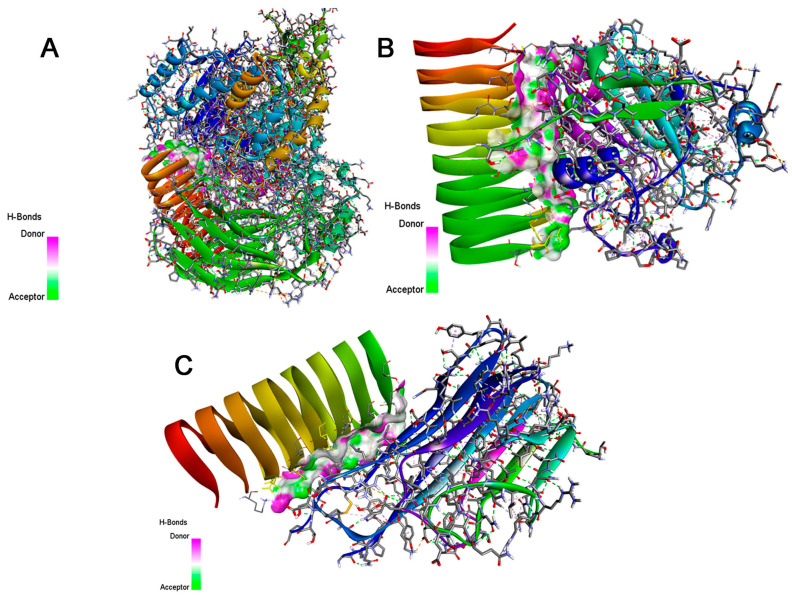
Three-dimensional illustrations exhibit protein-protein interactions of (**A**) sericin-PI3K, (**B**) sericin-GPx, and (**C**) sericin-TNF-α.

The interactions between SS (chain B) and three distinct proteins: PI3K (chain A), TNF-alpha (Chain A, C), and GPx (Chain A), demonstrate the pivotal role of hydrogen bonds and non-bonded contacts in stabilizing protein-protein interfaces. Using the PDBsum server (https://www.ebi.ac.uk/thornton-srv/databases/pdbsum/, accessed on 17 December 2024), we identified key residues at the interfaces of these complexes. In the PI3K-sericin complex, hydrogen bonds are formed between residues ASP50, ASN64, and ASP69 of sericin with ARG982 and ARG375 of PI3K, with bond distances ranging from 2.65 to 2.75 Å. Additionally, non-bonded contacts involving THR117 and LYS756 further reinforce the stability of the interface. Considering the interaction of sericin with GPx, the GPx-sericin complex is stabilized by hydrogen bonds between ASN49 and ASP170, and ASP50 and ARG175, while non-bonded interactions, such as those between LYS20 and SER61 from sericin with ALA192 and MET0 from GPX, further contribute to the molecular association. In the TNF-α-sericin interaction, hydrogen bonds between ASN35, ASP50, and ASN64 in sericin and GLU104, CYS101, and ARG103 in TNF-alpha are critical, with distances of 2.70 to 2.82 Å, complemented by non-bonded interactions that range from 2.72 to 3.66 Å. These contribute to the structural complementarity between the two proteins. These findings highlight the significant role of specific charged and polar residues in maintaining strong, stable protein-protein interactions. Silk sericin interactions with PI3K, GPx, and TNF-α were visualized using Discovery Studio software V 21.1.0 [54] as shown in Figure 13.

## 4. Discussion

This study seeks to assess the effectiveness of quercetin (QCT) and silk sericin (SS) from *T. alexandrinum* and silkworm *B. mori* in counteracting glucose and lipid metabolism disturbances in rats suffering from T2DM-induced liver impairment. Furthermore, we investigated the potential mechanisms by which QCT and SS abate hyperglycemia-induced dysregulation of hepatic gluconeogenesis and associated complications and orchestrate the PI3K/Akt/FOXO1 signaling pathway.

Our findings indicated amplified glucose and AGEs levels combined with a diminution in insulin concentrations in diabetic rats induced by Fructose/STZ. It is well known that induction with STZ impairs pancreatic β-cells, resulting in reduced activity and decreased insulin sensitivity, leading hyperglycemia [55]. Previous investigations revealed that induction with STZ elicits a surplus of ROS, impeding the electron transport chain in mitochondria, and resulting in decreased ATP synthesis and glucose detection loss from the secretion of insulin [56]. In addition, the polyol pathway, protein glycation, lipid peroxidation, and AGEs formation have been identified as potential factors contributing to the excessive generation of free radicals [57]. These complications reduce the body’s natural ability to counteract oxidation, engendering oxidative stress (OS) in the hepatic tissues.

Interestingly, supplementation with QCT and SS extracts enhanced glucose, AGEs, and insulin concentrations in diabetic animals. Consistent with our findings, prior investigations demonstrated that administering SS through the diet led to a lessening in plasma glucose and an expansion in insulin secretion following intraperitoneal glucose injection in rats fed a high-fat diet [58]. This could be explained by the hypoglycemic impact of QCT and SS through controlling enzymes responsible for glucose metabolism, modulating the functions of β-cells and insulin activity, and enhancing other parameters associated with diabetes [12,59].

The present study reports a critical disorder in MDA, SOD, GPx, and CAT, in addition to nitric oxide in hepatic tissues from Fructose/STZ-induced diabetic rats. This implies interference in the antioxidant defense system associated with expected inflammation. These observations are consistent with prior research, demonstrating that elevated MDA levels are indicative of increased oxidative damage to lipids in liver tissue [9]. In this context, it is shown that STZ poses organ damage in animals with DM and potentially triggers the oxidation of polyunsaturated fatty acids [60]. Critically, OS significantly contributes to the disruption of cellular activities in the liver and other organs, leading to increased vascular permeability and tissue damage [1]. Furthermore, hyperglycemia may trigger the generation of free radicals, resulting in DNA damage in addition to excessive production of nitric oxide, which functions as an inflammatory mediator that harms β-cells and reduces insulin synthesis [61,62]. In contrast, the enhancement of antioxidant parameters in hepatic tissues in QCT and SS-treated rats suggests that QCT and SS safeguard antioxidant mechanisms by precluding lipid peroxidation, thwarting oxidative DNA injury, and counteracting ROS in diabetic rats [60,63]. It is believed that the antioxidant activity of QCT as a flavonoid is derived from its chemical structure due to the presence of a hydroxyl group (OH) chiefly in the B and C rings, which contribute more than the A ring [15,64]. Additionally, in vivo investigations showed that QCT’s antioxidant property could be explained by its strong relationship with glutathione enzymatic activity, maintaining the oxidative balance inside cells [15]. On the other hand, previous studies reported that SS antioxidant features stem from the prevalence of polar amino acids, including serine, aspartic acid, threonine, and glutamic acid [11,27,65]. These amino acids bestow the SS with the remarkable capacity to scavenge free radicals, hindering OS within cells [27,66].

Considering lipid profiles, amplified levels of TC, TG, LDL-C, and VLDL-C, along with a remarkable drop in HDL-C level in Fructose/STZ-treated animals pointed out that a lack of insulin in individuals with DM causes various disruptions in metabolic and regulatory functions. Consequently, lipids accumulate in diabetic patients, leading to hypertriglyceridemia [67]. However, treatment with QCT and SS resulted in improved lipid profiles. These results match those of Biganeh et al. [68], demonstrating that SS ameliorated high-density lipoprotein, triglycerides, and total cholesterol in diabetic rats. Furthermore, QCT and SS had comparable beneficial effects on hepatic enzymes associated with lipid metabolism. Hence, our findings suggest that these agents possess advantageous properties for managing dyslipidemia linked to diabetic consequences [68,69]. 

Our results also showed that Fructose/STZ-induced diabetic rats had elevated ALT, AST, ALP, total bilirubin, and AFP, indicating that STZ incited hepatic damage and malfunction [70]. These findings are likely related to lipid peroxidation provoked by oxidative damage, which expands lysosomal membrane permeability and further enhances serum hepatocellular enzymes [71]. Furthermore, the high level of AFP in the blood is indicates hepatic impairment [72]. Our findings corroborate the observations of Uslu et al. [73], which demonstrated a substantial elevation of AFP expression in early T2DM. Besides, the diminution in the levels of total protein, albumin, and globulin in Fructose/STZ-induced diabetic rats corroborated the liver damage. A previous study reported that albumin and globulin function as transport proteins and biomarkers for pathological conditions [74]. By contrast, the improvement of these factors due to the administration of QCT and SS suggests their capacity to maintain the quality of plasma membranes, which in turn restores the levels of hepatic enzymes. In addition, Fructose/STZ-induced rats exhibited a substantial diminution in glycogen. According to Kuai et al. [75], diabetic patients have a reduced concentration of glycogen storage due to poor insulin-stimulated glycogen synthesis and dysregulation of glycogen metabolism. Moreover, the liver’s ability to activate glycogen synthase, which increases glycogen storage after a meal, is impaired in diabetic patients. This resulted in reduced glycogen, which is consistent with their food intake [8]. Conversely, diabetic rats receiving QCT and SS revealed a marked improvement in glycogen levels. This implies that both agents instigate pancreatic β-cells to increase insulin production, engendering the increase of glycogen in the liver of diabetic animals. This is achieved by stimulating glycogen synthase and inhibiting glycogen phosphorylase, resulting in an enhanced rate of glycogenesis [76,77].

It was reported that HK, GK, G6Pd, and G6P are crucial hepatic glucose-regulating enzymes to regulate glucose balance in the liver and the progression of DM [78]. The deficiency of these enzymes in DM patients may impede glycolysis. Our results revealed dysfunction in enzyme activity in Fructose/STZ-induced animals. Therefore, the drop in glycogen concentration in diabetic rats could be related to the diminution in HK and GK [6,79]. Critically, the reduction of these enzymatic activities engenders decreased glucose uptake in various organs, as well as the development of hyperglycemia [80]. Intriguingly, Intriguingly, QCT and SS effectively restore the levels of HK, GK, G6Pd, and G6P. Their capacity to improve hyperglycemia may be ascribed to their modulation of hepatic enzymes implicated in glycogen synthesis, glycolysis, and gluconeogenesis. Accordingly, they contribute to the restoration of insulin concertation to normal in mice with T2DM [81,82].

Considering inflammation in hepatic tissues, our findings indicated that Fructose/STZ diabetic rats displayed heightened TNF-α, IL-1β, IL-6, and COX-2 levels. These results are supported by prior reports, which revealed that OS development as a result of STZ induction interacts with β-cells, disordering signaling pathways, and increasing proinflammatory markers [12,83]. Importantly, the administration of QCT and SS to diabetic rats precluded the generation of harmful cytokines linked to diabetes onset and progression. Recent investigations demonstrated the anti-inflammatory properties of QCT and SS [1,84]. It has been proven that the inhibition of COX-2 and nitric oxide genes expression is one of the anti-inflammatory mechanisms of QCT and SS [85,86]. Furthermore, the potency of SS to diminish the levels of inflammatory mediators, including TNF-α, IL-6, and IL-1β, mitigate the inflammation and its associated consequences [85,87,88].

Our study also reported notable downregulations in hepatic Akt, PI3K, and FOXO1 expressions in Fructose/STZ-diabetic rats compared with control animals. These findings are supported by earlier observations, which evinced lower hepatic Akt, PI3K, and FOXO1 gene expressions in diabetic rat livers [83,89]. In insulin signaling, the PI3K/p-Akt/FOXO1 pathway regulates blood sugar, while the PI3K/Akt pathway controls hepatic glucose uptake. FOXO1, a key downstream factor, is liberated from the nucleus upon Akt phosphorylation, inhibiting gluconeogenic genes, phosphoenolpyruvate carboxykinase (PEPCK), and G-6pase. Additionally, AMPK, another regulator, coordinates metabolism in T2DM and influences inflammation and oxidative stress. A previous study found that SS stimulates glycogen breakdown by controlling PI3K/Akt activity and the phosphorylation of FOXO1 protein [90]. Insulin binds to IR on the liver’s cell membrane, activating the insulin receptor substrate, which further activates PI3K. As a result, PI3K produces a second messenger that stimulates AKT. A previous study substantiated that the p-Akt pathway hinders the glucose metabolism of hepatocytes [91]. Interestingly, QCT and SS effectively restore PI3K/Akt/FOXO1 genes expression, indicating their anti-diabetic effects via enhancing insulin receptor-mediated signaling to modulate blood sugar levels [82,92]. This suggests a potential role for QCT and SS in modulating the liver’s insulin-PI3K/Akt pathway. Also, QCT and SS may promote glycolysis and glycogen synthesis while suppressing gluconeogenesis and lipogenesis. Additionally, they could improve insulin sensitivity and glucose uptake.

These biochemical parameters are corroborated by histological examinations, which exhibited hepatocellular impairments in diabetic rats. Similar observations were reported, including disorganized architecture, central vein congestion, and cellular alterations like pyknosis and vacuolization, which confirmed hepatocellular degeneration [93]. Furthermore, the observed steatosis suggests dysregulated lipid metabolism with anomalous fat deposition, which is a key factor in impaired liver disease [94]. In contrast, Fructose/STZ-diabetic rats receiving QCT and SS evinced a remarkable restoration of normal histological features of hepatic tissues. This could be ascribed to the boosting of antioxidant enzymes and elimination of harmful radicals such as hydroxyl, superoxide, alkoxyl, and peroxyl radicals [63].

Furthermore, immunohistochemical analysis revealed that Fructose/STZ diabetic rats had higher caspase-3 immune-reactive cell expression than control rats. These results match those reported in previous immunocytochemistry-based studies [95]. It is suggested that long-term high blood sugar levels lead to changes in the pro-apoptotic BAX and anti-apoptotic Bcl-2 proteins, while also elevating the expression of p53, caspase-8, and caspase-9 through the mitochondrial apoptosis pathway. These activities stimulate caspase-3, which regulates cell apoptosis [96]. Conversely, administration of QCT or SS to diabetic rats remarkably dwindled caspase-3 expression, preventing hepatic cell death [5,97].

The in silico findings indicated that QCT demonstrated superior antidiabetic effects than SS. The stronger binding affinity of QCT to PI3K, GPx, and TNF-α suggests that QCT may attenuate oxidative stress and inflammation and regulate hepatic gluconeogenesis more than SS in diabetic rats. This aligns with experimental results, highlighting potential effect of QCT as a potent therapeutic agent in diabetes management. Despite the marked interactions of SS with PI3K, GPx, and TNF-α, the lower HADDOCK scores and less favorable docking parameters suggest that it has binding less stable and weaker than QCT. Although these findings imply that SS possesses potential efficacy in modulating the PI3K/Akt/FOXO1 signaling pathway and attenuating hepatic gluconeogenesis, it may be less potent than quercetin. In silico findings indicate that quercetin’s molecular interactions are likely more robust and biologically relevant for achieving superior antidiabetic effects than SS. Altogether, our results accentuated the vital role of QCT and SS as anti-diabetic agents by potentially orchestrating the PI3K/Akt/FOXO1 signaling pathway with better performance of QCT.

## 5. Limitations and Future Prospects

Although this study provides compelling evidence about the antidiabetic activities of QCT and SS employing in vivo and in silico examinations, future studies are necessary to better comprehend their underlying mechanisms at the molecular and cellular levels. In this context, western blot and immunohistochemical analyses, along with further proteomic and transcriptomic studies, should be carried out. Moreover, future investigations are warranted to design drug delivery systems for QCT and SS utilizing various polymeric carriers to target specific sites, sustain their activities, and govern drug release.

## 6. Conclusions

In conclusion, this paper evaluated the potential application of quercetin (QCT) and silk sericin (SS) from *T. alexandrinum* and silkworm *B. mori* for alleviating hyperglycemia-induced hepatic gluconeogenesis dysregulation and restoring hepatic function in diabetic rats. Notably, QCT and SS treatments demonstrated anti-diabetic, antioxidant, and anti-inflammatory properties. Our findings revealed that QCT and SS improved insulin sensitivity, normalized blood glucose levels, enhanced glycogen storage, suppressed hepatic gluconeogenesis, and improved liver function. Additionally, QCT and SS therapies resulted in a favorable lipid profile and attenuated oxidative stress and inflammatory biomarkers in the liver, abating cellular dysfunction. Considering signaling pathways, the results demonstrated that QCT and SS potentially orchestrated the PI3K/Akt/FOXO1 signaling pathway. This resulted in the reinstatement of normal hepatic tissue architecture as perceived in histopathological examination. Most notably, immunohistochemical analyses exhibited that both therapies precluded cellular apoptosis given the marked reduction in caspase-3 expression. The in silico investigations substantiated the biological findings. Collectively, these outcomes point toward the potential role of QCT and SS in managing diabetes by modulating hepatic glucose production via the PI3K/Akt/FOXO1 pathway.

## Figures and Tables

**Table 1:** Predicted binding site of the studied proteins.

Structure	Chain ID	Active Site Residues
Sericin (3ULT)	B	34, 35, 49, 50, 52, 61, 62, 63, 64, 66, 75, 76, 77, 78, 80, 90, 92, 94, 106, 107, 108
PI3K (1E90)	A	473, 474, 475, 477, 525, 526, 527, 528;898, 946, 948, 950, 951, 963, 964, 968, 980, 1081, 1087, 1085, 1086, 1088, 1089, 1090, 1091, 1092, 1093, 1094
GPx (2F8A)	A	−1, 0, 12, 13, 125, 156, 129, 130, 131, 170, 172, 179, 174, 175
TNF-α (6OP0)	A	75, 77, 95, 96, 97, 98, 99, 102, 115, 137

**Table 2:** Quantification of lesions observed in histological sections of liver tissues stained with H&E.

Lesions	Experimental Groups			
	Control	Diabetic	Diabetic + Quercetin	Diabetic + Silk Sericin
Fatty degeneration	5.7 ± 2.1 ^a^	40 ± 3.1 ^b^	6.3 ± 2.3 ^a^	8.7 ± 2.1 ^a^
Pyknotic nuclei	2.3 ± 2.1 ^a^	29 ± 1.7 ^b^	4.3 ± 0.6 ^a^	4.3 ± 1.5 ^a^
Dilatation of sinusoidal vasculature	1.6 ± 0.6 ^a^	13 ± 3.1 ^b^	2.0 ± 1.0 ^a^	2.3 ± 1.5 ^a^
Kupffer cells	15 ± 0.6 ^a^	25 ± 1.2 ^b^	18 ± 2.0 ^a^	17 ± 2.3 ^a^
Infiltration of inflammatory cells	5.7 ± 1.5 ^a^	185 ± 13 ^b^	5.7 ± 2.5 ^a^	7.3 ± 3.5 ^a^
Ballooning degeneration	2.7 ± 1.5 ^a^	42 ± 5.7 ^b^	5.0 ± 2.6 ^a^	4.0 ± 1 ^a^
Giant nuclei	1.7 ± 0.6 ^a^	4.7 ± 0.6 ^b^	1.7 ± 0.6 ^a^	2.0 ± 1 ^a^

All values are presented as mean ± SD at *p* ≤ 0.05. Different letters indicate significant differences between groups. Quantification was performed using three slides from each group represented by three animals.

**Table 3:** Docking results of QCT with target proteins.

Protein	Binding Energy (kcal/mol)	Docking Center (Å)	Coordinates (X, Y, Z)
PI3K (1E90)	−8.1	(X = 22.06, Y = 61.51, Z = 21.79)	X = 22.06, Y = 61.51, Z = 21.79
GPx (2F8A)	−6.4	(X = 15.49, Y = 19.50, Z = 28.35)	X = 15.49, Y = 19.50, Z = 28.35
TNF-α (6OP0)	−10.3	(X = −12.09, Y = −0.86, Z = 17.83)	X = −12.09, Y = −0.86, Z = 17.83

**Table 4:** HADDOCK results of silk sericin docked to target proteins.

Protein	HADDOCK Score (kcal/mol)	RMSD from Lowest-Energy Structure (Å)	Van der Waals Energy (kcal/mol)	Electrostatic Energy (kcal/mol)	Desolvation Energy (kcal/mol)	Z-Score
PI3K (1E90)	−69.3	0.5 ± 0.3	−62.3 ± 7.4	−330.0 ± 31.0	1.3 ± 4.8	−1.3
GPx (2F8A)	−75.3	0.6 ± 0.3	−47.9 ± 3.6	−217.6 ± 23.5	7.7 ± 2.4	−2.0
TNF-α (6OP0)	−60.4	0.6 ± 0.6	−46.7 ± 3.7	−132.0 ± 30.5	3.6 ± 3.0	−1.4

## Data Availability

The datasets generated during the current study are available from the corresponding authors upon reasonable request.

## Abbreviations

T2DM: Type 2 diabetes mellitus; PI3K: phosphatidylinositol 3-kinase; p-Akt: phospho-Akt or protein kinase B; FOXO1: forkhead box-O1; TC: Total cholesterol; TG: Triglycerides; GK: Glucokinase; HK: Hexokinase; G6Pd: Glucose-6-phosphate dehydrogenase; G6P: Glucose-6-phosphate; AGEs: advanced glycation end-products; AST: Aspartate aminotransferase; ALT: alanine transaminase; ALP: alkaline phosphatase; AFP: Alpha-fetoprotein; MDA: Malondialdehyde; GSH: Reduced glutathione; SOD: Superoxide dismutase; CAT: catalase activity; GPx: glutathione peroxidase; TNF- α: tumor necrosis factor alpha; IL-1β: interleukin-1β; IL-6: interleukin-6; COX-2: cyclooxygenase-2.
